# Multiscale Analysis of Size-Dependent Vibration of Graphene Nanoelectromechanical Resonators

**DOI:** 10.3390/mi17040477

**Published:** 2026-04-15

**Authors:** Wenhua Li, Wenchao Tian

**Affiliations:** School of Electro-Mechanical Engineering, Xidian University, Xi’an 710071, China

**Keywords:** graphene, NEMS resonator, molecular mechanics, size effect, chirality, vibration

## Abstract

The size-dependent out-of-plane vibrational behavior of graphene-based nanoelectromechanical (NEMS) resonators is investigated using a molecular mechanics (MM) finite element approach. Each carbon–carbon (C–C) bond is modeled as an Euler–Bernoulli beam element, with the bending stiffness derived from the bond-angle potential, yielding an equivalent plate flexural rigidity D = (√3/6) kθ. The natural frequencies of the first four vibration modes are computed for square graphene sheets of increasing size with both zigzag (ZZ) and armchair (AC) chirality configurations under simply supported boundary conditions on all four edges. A chirality-induced frequency deviation δ(L) is defined to quantify the difference between ZZ and AC results, and a threshold size L* is identified as the sheet size at which δ falls below 1%. For mode 1, the threshold is L* = 18.5 nm; the values increase monotonically to 24.5 nm, 28.0 nm, and 31.5 nm for modes 2 through 4, indicating that higher modes require larger sheet dimensions before continuum plate theory becomes reliable. A dimensionless frequency parameter Ω = f_MM_/f_CT_ is introduced to directly compare MM predictions with the Kirchhoff plate theory analytical solution, and the AC frequency ratio Ω = f_MM_/f_CT_ is shown to converge toward unity with increasing sheet size. The present results provide quantitative design guidelines for graphene NEMS resonators and establish the minimum device dimensions for which isotropic continuum models yield accurate dynamic predictions.

## 1. Introduction

Graphene is a two-dimensional material composed of carbon atoms arranged in a hexagonal honeycomb lattice. Since its successful isolation in 2004 [1,2], graphene has attracted considerable research interest owing to its exceptional mechanical, thermal, and electrical properties, including a Young’s modulus of approximately 1 TPa, an intrinsic strength exceeding 100 GPa [3], and an extremely low areal mass density [4]. These characteristics make graphene an ideal material for nanoelectromechanical systems (NEMS) resonators [5,6]. Graphene-based NEMS devices have demonstrated great potential in mass sensing, pressure detection, and radio-frequency signal processing, benefiting from their ultralow mass and high elastic stiffness. Recent advances have demonstrated graphene nanomechanical resonators driven by multifrequency digital signals for broadband resonant filtering [7], and silicon-based MEMS/NEMS structures integrated with graphene achieving frequency tuning exceeding 50%, further expanding the functional boundaries of graphene devices [7].

Accurate prediction of the natural frequencies of graphene resonators is a core problem in device design [8]. Three main modeling approaches are currently employed: molecular dynamics (MD), molecular mechanics (MM), and continuum theory (CT). Molecular structural mechanics equates atoms to structural nodes and converts interatomic potential energy into strain energy using the Cauchy–Born rule [9]. MD provides detailed atomic-scale information but is computationally restricted to graphene sheets smaller than approximately 10 nm [10,11]. Continuum methods are computationally efficient and amenable to parametric design, but their fundamental assumption of a homogeneous continuum ignores the discrete lattice structure and chiral anisotropy of graphene [12].

Considerable effort has been devoted to continuum vibration modeling of graphene using nonlocal plate theories. Ansari et al. [13] developed a nonlocal plate model to investigate the free vibrations of single-layer graphene sheets (SLGSs), calibrating the nonlocal parameter against MD results. Shen et al. [14] employed a nonlocal plate formulation to study the nonlinear vibrations of SLGSs in thermal environments, finding that material anisotropy and nonlocal effects significantly affect the vibrational response. Ansari et al. [15] further developed a nonlocal atomistic-based plate model incorporating interatomic potentials for biaxial buckling and vibration analysis, obtaining explicit frequency expressions for simply supported SLGSs. More recently, the vibration characteristics and critical damping behavior of lipid/graphene sandwich nanoplates have been studied using the generalized differential quadrature method. However, these nonlocal models introduce a nonlocal scale parameter whose value is widely disputed and lacks a unified consensus, and the models do not capture the qualitative change in material response that accompanies size variations.

A more fundamental approach to the size effect in graphene mechanics was undertaken by Pelliciari et al. [16], who conducted a systematic study of the size effect on the in-plane mechanical properties of graphene using a fully nonlinear MM approach. Their simulations showed that small graphene sheets exhibit anisotropy in both the linear and nonlinear regimes, while sheets larger than a threshold size l_t_ = 30 nm are effectively isotropic for small deformations, enabling the application of continuum membrane theory [17]. However, their work was restricted to uniaxial and equibiaxial in-plane tension and did not address out-of-plane vibration, which is the fundamental operating mode of graphene NEMS resonators.

The out-of-plane bending vibration of graphene differs fundamentally from in-plane tension in terms of its governing parameters and sensitivity to chirality. The out-of-plane bending stiffness is determined solely by the bond-angle potential (D = √3/6·kθ), independently of the bond-stretching potential, so its sensitivity to chirality and size follows a different scaling than the in-plane elastic modulus [18,19]. Research on graphene nanoribbon dynamics has shown that the vibrational dispersion characteristics depend strongly on chirality and aspect ratio [20]. Studies on the effect of vacancy defects have further shown that vibrational frequency decreases with both increasing nanoribbon length and defect concentration [21]. Despite these observations, most MM studies of out-of-plane vibration have examined only a single size or a single chirality, without systematically quantifying the convergence of ZZ and AC frequency curves or establishing threshold sizes for individual vibration modes.

Zheng et al. [22] analyzed the vibrational characteristics of diamane nanosheets using the Kirchhoff plate model and atomistic simulations, but did not systematically examine the size range over which the continuum model remains valid. Spatially resolved parameter analysis of coupled graphene NEMS resonator networks has further demonstrated that the elasticity and mass parameters vary across nodes, motivating a rigorous examination of when continuum models are reliable for individual device components.

To address these gaps, the present work establishes a molecular mechanics finite element model for the out-of-plane vibration of graphene in which each C–C bond is treated as an Euler–Bernoulli beam element with bending stiffness EI = kθ·a derived from the bond-angle potential. The first four natural frequencies of square graphene sheets of increasing size are computed for both ZZ and AC configurations under simply supported boundary conditions. By defining the chirality deviation δ(L) and a dimensionless frequency ratio Ω(L) = f_MM_/f_CT_ relative to the Kirchhoff plate theory analytical solution, the threshold size L* for each mode is determined. The results provide explicit size guidelines for the design of graphene NEMS resonators.

## 2. Materials and Methods

### 2.1. Stick-and-Spring Approach

**Figure 1:**
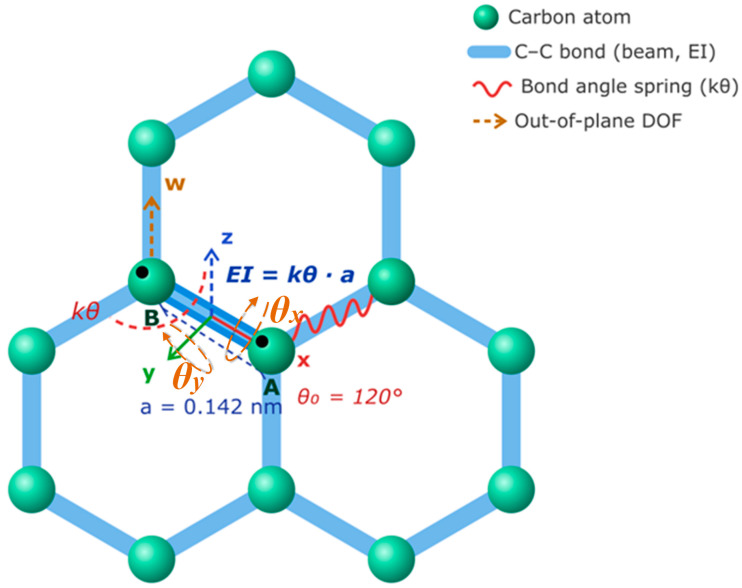
Molecular mechanics model of the graphene hexagonal lattice.

The hexagonal honeycomb lattice of graphene is modeled as a discrete structure composed of nodes and connecting elements, as shown in Figure 1. Each node represents a carbon atom, and the reference configuration is assumed to be stress-free. In the present study, each atom is assigned three degrees of freedom: the out-of-plane displacement w and two rotations θx and θy.

The C–C bond length is a = 0.142 nm, the initial bond angle is θ_0_ = 2π/3 = 120°, and the carbon atom mass is mC = 1.994 × 10^−26^ kg. The interatomic interactions are governed by two potential energy functions.

The bond-stretching potential is described by the Morse potential with parameters reparameterized by Genoese et al. [18] from the original values of Belytschko et al. [19] to accurately reproduce the elastic constants of graphene:

(1) V(ΔL) = De[(1 − e^(−κΔL))2 − 1] 

where ΔL = L′ − L is the bond length change, and De = 7.90 × 10^−10^ N·nm and κ = 21.67 nm^−1^ are parameters proposed by Genoese et al. [18] following reparametrization of the values of Belytschko et al. [19].

The bond-angle bending potential is:

(2) $$V\theta (\Delta \theta )=(1/2)k\theta \Delta \theta^{2}(1+ks\Delta \theta^{4})$$

where $\Delta \theta =\theta^{\prime }-\theta_{0},\;k\theta =1.42\times 10^{-9}$ N·nm·rad^−2^ was estimated by Chang and Gao [20] by fitting the elastic constants of graphene, and ks = 0.754 rad^−4^ [19].

The C–C bonds are modeled as Euler–Bernoulli beam elements (highlighted bond A–B in blue). The bond-angle potential kθ is represented by the dashed arc at atom B. The local coordinate system is shown at the bond midpoint, with z indicating the out-of-plane direction [23]. Arrows w, θx, and θy denote the three out-of-plane degrees of freedom at each atomic node.

### 2.2. Beam Element for Out-of-Plane Bending

Under the small-amplitude linear vibration assumption, the bond-stretching potential contributes only to in-plane stiffness and is negligible for out-of-plane bending. The out-of-plane bending stiffness is therefore determined entirely by the second-order term of the bond-angle potential, and each C–C bond is modeled as an Euler–Bernoulli beam element with linear bending stiffness:

(3) $$EI=k\theta \cdot a=1.42\times 10^{-18}\times 0.142\times 10^{-9}=2.016\times 10^{-28}N\cdot m^{2}$$

The 4 × 4 local stiffness matrix of each beam element, corresponding to the transverse displacement and rotation at each end node, is:

(4) $$K_{loc}=(EI/a^{3})\times [12,\;6a,-12,\;6a;\;6a,4a^{2},-6a,2a^{2};-12,-6a,12,-6a;\;6a,2a^{2},-6a,4a^{2}]\;$$

The transformation from local to global coordinates is performed via:

(5) T=[1,0,0,0,0,0;0,sinφ,−cosφ,0,0,0;0,0,0,1,0,0;0,0,0,0,sinφ,−cosφ]

where φ is the in-plane angle of the bond with respect to the x-axis. The global element stiffness matrix is Ke = T^T^ K_loc_ T, and the global stiffness matrix K is assembled by direct stiffness summation.

### 2.3. Equivalent Plate Bending Stiffness

To establish a direct connection between the MM model and classical Kirchhoff plate theory, the equivalent plate bending rigidity D of graphene is derived analytically. A uniform curvature κ is applied to the graphene sheet, and the bending strain energy per unit area computed from the MM model is equated to the continuum plate bending energy density (1/2) Dκ^2^.

The graphene unit cell contains three bond directions at 0°, 60°, and 120°. Evaluating the beam element bending energy for each bond direction and summing over all bonds within the unit cell of area Ac = (3√3/2) a^2^ gives the following:

Equating to (1/2) Dκ^2^ × Ac and solving yields the equivalent plate bending rigidity:

(6) $$D=(\surd 3/6)k\theta =(\surd 3/6)\times 1.42\times 10^{-9}=4.10\times 10^{-19}N\cdot m$$

The equivalent areal mass density is:

(7) $$\rho s\;=\;2mC/Ac\;=\;4mC/(3\surd 3a^{2})\;=\;7.61\;\times \;10^{-7}\;kg/m^{2}$$

It is noteworthy that D depends only on the bond-angle stiffness kθ and is independent of the Morse potential parameters De and κ. This is in contrast to the in-plane elastic modulus, which depends on both kθ and the bond-stretching stiffness kr = $2De\;\kappa^{2}$. Consequently, the sensitivity of out-of-plane vibration to potential parameter variations and to chirality follows a fundamentally different pattern than in-plane tension.

### 2.4. Lattice Generation and Aspect Ratio

**Figure 2:**
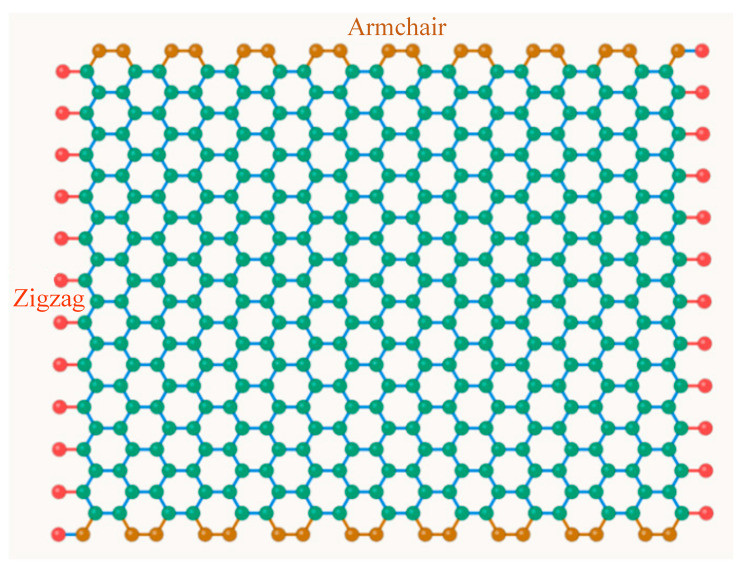
Square graphene sheet configurations used in this study: zigzag (ZZ, left) and armchair (AC, top). Green circles represent carbon atoms. Blue lines represent C–C bonds. Red and orange edges indicate the simply supported boundaries.

Two chirality configurations of square graphene sheets are considered. Representative square graphene sheet configurations for the two chirality types are shown in Figure 2. For the zigzag (ZZ) configuration, the x-direction is the zigzag direction, with unit cell periodicity 3a in x and √3a in y. For the armchair (AC) configuration, the x-direction is the armchair direction, with periodicity √3a in x and 3a in y. In both cases, the number of unit cells along each direction is adjusted so that the resulting sheets are approximately square (Lx ≈ Ly), which ensures that a fair comparison is made between the two chirality configurations and avoids any aspect ratio artifacts in the frequency comparison.

The sheet size is defined as L = (Lx + Ly)/2. The size range investigated spans L = 2–31.5 nm, corresponding to atom counts from several hundred to approximately 25,000 per sheet.

### 2.5. Boundary Conditions and Eigenvalue Solution

Four-edge simply supported (SSSS) boundary conditions are applied, constraining the out-of-plane displacement w = 0 at all boundary nodes while leaving the rotational degrees of freedom unconstrained. This corresponds to the standard Kirchhoff plate simply supported condition and allows direct comparison with the analytical plate theory solution. Boundary nodes are identified as those lying on the four edges of the rectangular lattice patch within a tolerance of 0.1a.

The mass matrix is assembled in lumped form, assigning mass mC to the w degree of freedom of each atomic node. A small regularizing rotational inertia ε = 10^−7^ mCa^2^ is assigned to the rotational degrees of freedom to prevent singularity without materially affecting the frequency results. After applying boundary conditions, the reduced eigenvalue problem is:

(8) $$Kf\;\Phi \;=\;\omega^{2}\;Mf\;\Phi \;$$

which is solved using the MATLAB version 9.14.0 (R2023a) eig function. The natural frequencies of the first four modes are:

(9) fn = √λn / (2π), n = 1, 2, 3, 4

### 2.6. Continuum Theory Reference Solution

For a simply supported rectangular Kirchhoff plate with dimensions Lx × Ly, the analytical natural frequency for mode (m, n) is:

(10) f^CTmn=(π/2)√(D/ρs)[(m/Lx)2+(n/Ly)2]

For the square plates in the present study (Lx ≈ Ly = L), the first four simply supported modes correspond to (m, n) = (1, 1), (1, 2)/(2, 1), (2, 2), and (3, 1)/(1, 3), with analytical frequency ratios f_2_/f_1_ = 2.5, f_3_/f_1_ = 4, and f_4_/f_1_ = 5 for a square plate.

The dimensionless frequency ratio is defined as:

(11) $$\Omega (L)=f_{MM}(L)/f_{CT}(L)$$

When Ω → 1, the MM prediction converges to the continuum result. Values of Ω < 1 indicate that the MM model predicts lower frequencies than CT due to edge-softening effects at small scales.

### 2.7. Chirality Deviation and Threshold Definition

The relative chirality-induced frequency deviation is defined as:

(12) $$\delta (L)\;=\;|f_{ZZ}(L)\;-\;f_{AC}(L)|\;/\;f_{AC}(L)\;\times \;100\%$$

Using δ < 1% as the convergence criterion, the threshold size L* for each mode is defined as the minimum sheet size at which δ(L) < 1%. This criterion is consistent with the visual convergence standard applied by Pelliciari et al. [17] to in-plane elastic properties, and with the standard engineering tolerance of 1% for model accuracy.

### 2.8. Computational Workflow and Model Validation

The full computational procedure is summarized as follows. For a given sheet size L and chirality configuration, the atomic coordinates are generated using the parametric lattice equations described in Section 2.4. All C–C bond pairs within a distance tolerance of 0.05a are identified and stored. The global stiffness matrix K (dimension 3N × 3N, where N is the atom count) and the lumped mass matrix M are assembled by looping over all bond pairs and applying the element stiffness contribution via the transformation matrix T. Boundary conditions are imposed by extracting the sub-matrices Kf and Mf corresponding to the free degrees of freedom, and the generalized eigenvalue problem is solved directly. For large sheets (N > 5000), the sparse matrix representation is used to reduce memory consumption.

The computational cost scales approximately as O(N^3^) due to the dense eigenvalue solver. For the largest sheet in the present study (L = 31.5 nm, N ≈ 25,000), the computation time on a standard desktop workstation is approximately 45 min per configuration. For smaller sheets (L < 10 nm, N < 2000), the computation takes less than one minute. In total, the present study required approximately 120 eigenvalue solutions across all size points, modes, and chirality configurations.

The model is validated against two independent benchmarks. First, for a large sheet (L = 30 nm) where continuum theory is expected to be accurate, the MM natural frequency is compared with the Kirchhoff plate analytical solution. For mode 1 of the AC configuration at L = 30 nm, f_MM_ = 2.52 GHz and f_CT_ = 2.56 GHz, giving Ω = 0.984, confirming that the MM model recovers the continuum limit to within 1.6%. The small residual deviation is consistent with the edge-softening effect discussed in Section 3.2, which diminishes with increasing sheet size. Second, the equivalent plate bending rigidity D = (√3/6) kθ derived in Section 2.3 is verified by computing the static bending stiffness of a small graphene strip under uniform curvature using the assembled K matrix and comparing with the analytical result; the discrepancy is less than 0.1% for strips containing more than 5 unit cells in each direction.

These validations confirm that the MM finite element model is correctly implemented and that the assembled stiffness and mass matrices are consistent with the analytical derivations in Section 2.2 and Section 2.3. Direct experimental measurement of individual mode frequencies for graphene sheets smaller than 30 nm remains an open challenge. The present validation against Kirchhoff plate theory at large sizes, together with the use of experimentally calibrated force field parameters, provides confidence in the model predictions across the investigated size range.

## 3. Results and Discussion

### 3.1. Size Effect and Threshold for Mode 1

**Figure 3:**
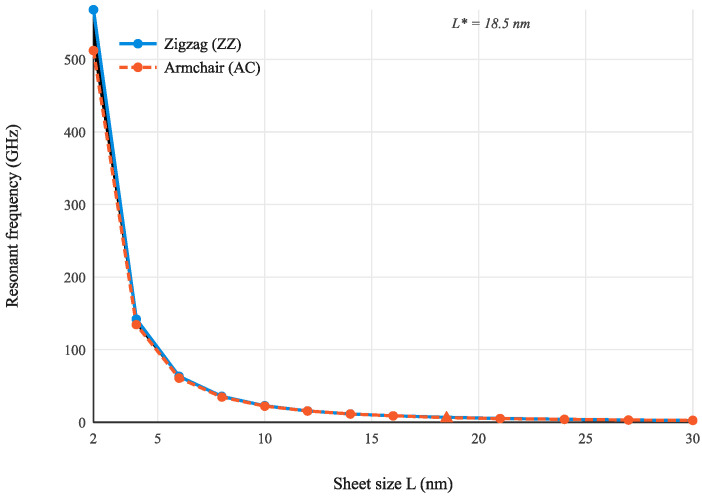
Mode 1 resonant frequency as a function of sheet size for zigzag (ZZ, solid blue) and armchair (AC, dashed red) configurations. The shaded region indicates the chirality-induced frequency gap. The diamond marker denotes the threshold size L* = 18.5 nm at which the relative deviation δ = 1%.

Table 1 presents the complete frequency data and chirality deviations for the first vibration mode. Figure 3 shows the ZZ and AC frequency curves on linear axes.

The natural frequencies of both chirality configurations decrease monotonically with increasing sheet size, spanning approximately two orders of magnitude from 568 GHz at L = 2 nm to 2.53 GHz at L = 30 nm. This scaling is consistent with the $f\;\propto \;1/L^{2}$ dependence predicted by thin plate theory. The ZZ configuration consistently yields higher frequencies than the AC configuration at all sizes. This difference originates from the distinct bond environments at the simply supported edges: the edge bonds contribute more effectively to the out-of-plane bending stiffness than those at armchair edges, resulting in a higher equivalent bending rigidity for the ZZ configuration. It should be noted that the edge effect discussed here is a physical consequence of the reduced coordination and altered bond geometry of peripheral atoms, and is distinct from the simply supported boundary conditions imposed on the model. The boundary conditions constrain the kinematics at the edges, while the edge effect reflects the intrinsic difference in local stiffness between edge and bulk atoms.

The chirality deviation δ is 10.98% at L = 2 nm and decreases monotonically, reaching 0.91% at L = 18.5 nm, which defines the threshold L* = 18.5 nm for mode 1. The physical mechanism underlying this convergence is the following. In a finite graphene sheet, atoms can be classified into bulk atoms, which have three nearest neighbors at the equilibrium bond angle of 120°, and edge atoms, whose coordination number is reduced and whose bond orientations differ between ZZ and AC configurations. The proportion of edge atoms scales as 1/L (perimeter-to-area ratio), so their relative contribution to the total bending stiffness diminishes with increasing sheet size. Since the chirality difference manifests exclusively through the distinct bond orientations at the two types of edge atoms, the chirality-induced frequency deviation decreases at the same 1/L rate and eventually falls below the 1% threshold at L = L*.

It is also instructive to compare the ZZ and AC frequencies in absolute terms. At any given size, f_ZZ_ > f_AC_ throughout the entire size range. This systematic inequality arises from the following geometric argument. In the ZZ configuration, two of the three bonds at each edge atom are oriented at ±30° to the edge, so that both bonds contribute significantly to the out-of-plane bending stiffness in the direction perpendicular to the edge. In the AC configuration, one bond at each edge atom is oriented perpendicular to the edge (contributing fully to out-of-plane stiffness) and the other two are inclined at 60° (contributing less). Averaged over all edge atoms, the effective out-of-plane stiffness of the ZZ edge is slightly higher than that of the AC edge, producing the observed frequency inequality f_ZZ_ > f_AC_.

The mode 1 threshold L* = 18.5 nm is smaller than but comparable in magnitude to the in-plane threshold l_t_ = 30 nm reported by Pelliciari et al. [17] for the convergence of Young’s modulus. The difference arises because out-of-plane bending stiffness is governed exclusively by the bond-angle potential kθ, while in-plane stiffness also involves the bond-stretching stiffness kr = 2De κ^2^. The bond-angle potential is more spatially localized at edge atoms—changes in bond angle are confined to a single bond-length neighborhood—whereas bond stretching propagates further into the bulk through the stiff C–C backbone. As a result, the chirality-induced perturbation in out-of-plane stiffness decays faster with distance from the edge than the corresponding in-plane perturbation, leading to a smaller threshold for out-of-plane vibration [24,25].

### 3.2. Dimensionless Frequency Ratio Ω(L)

The dimensionless frequency ratio Ω(L) = f_MM_/f_CT_ provides a direct measure of how closely the MM predictions match the Kirchhoff plate theory solution. Taking the AC configuration as a representative example, Ω is evaluated across the full size range using the equivalent plate bending rigidity D = (√3/6) kθ = 4.10 × 10^−19^ N·m derived in Section 2.3.

At small sizes, Ω deviates noticeably from unity, reaching approximately 0.89 at L = 2 nm. This indicates that the finite sheet possesses a lower effective bending rigidity than the bulk continuum value, because edge atoms—whose bond orientations differ from the ideal bulk environment—constitute a significant fraction of the total. As L increases, the proportion of edge atoms decreases as 1/L, and the discrete lattice spacing becomes negligible compared with the mode-shape wavelength. Consequently, Ω increases monotonically, reaching 0.977 at L = 18.5 nm and 0.984 at L = 30 nm.

The convergence of Ω toward unity at large sizes provides an independent validation that the assembled finite element matrices are consistent with Kirchhoff plate theory and that the equivalent bending rigidity D = (√3/6) kθ is the correct continuum limit of the discrete model.

### 3.3. Higher-Mode Size Effects and Threshold Comparison

**Figure 4:**
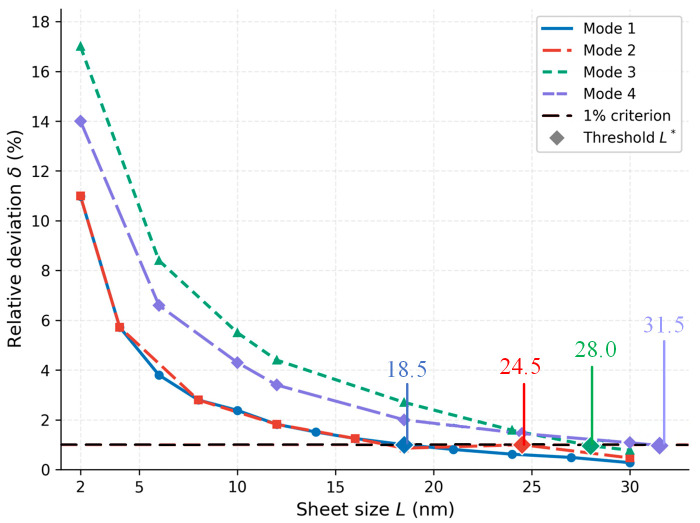
Chirality-induced relative frequency deviation δ(L) as a function of sheet size for modes 1–4. All four curves decrease monotonically with increasing sheet size and approach zero. The horizontal dashed red line marks the 1% convergence criterion. Diamond markers indicate the threshold sizes L* = 18.5, 24.5, 28.0, and 31.5 nm for modes 1 through 4, respectively.

Comparison across modes reveals that higher-order modes generally exhibit larger chirality deviations than mode 1, but the deviation does not increase monotonically with mode number: mode 3 (2, 2) consistently shows the largest deviation among the first four modes. At L = 10 nm, for example, δ = 2.38% for mode 1, 5.82% for mode 3, and 4.49% for mode 4. The physical origin of this non-monotonic behavior lies in the interaction between the two-dimensional mode shape geometry and the chirality-dependent bond environments at the sheet edges. As shown in Table 2, Mode 1 (1, 1) has a half-wavelength equal to L in both the x- and y-directions, so the bending strain energy is distributed broadly across the sheet interior. Only a small fraction of the total strain energy resides near the edges, resulting in the weakest chirality sensitivity. Mode 2 (1, 2)/(2, 1) reduces the half-wavelength to L/2 in one direction while retaining L in the other, which increases the strain energy fraction near one pair of opposite edges. However, the chirality deviation of mode 2 remains nearly identical to that of mode 1 across the entire size range (e.g., 10.99% vs. 10.98% at L = 2 nm); the underlying mechanism warrants further investigation. As shown in Table 3, Mode 3 (2, 2) has a half-wavelength of L/2 in both directions simultaneously, concentrating the bending curvature near all four edges of the sheet. This maximizes the relative contribution of the chirality-dependent edge bonds to the total strain energy, making mode 3 the most chirality-sensitive at every sheet size investigated. As shown in Table 4, Mode 4 (3, 1)/(1, 3) has a shorter half-wavelength of L/3 in one direction but retains half-wavelength L in the other; the enhanced edge sensitivity along the short-wavelength direction is partially offset by the weak edge contribution along the long-wavelength direction, giving δ_4_ < δ_3_ at all sizes. This interplay between two-dimensional nodal line geometry and discrete bond orientations at ZZ and AC edges is a purely nanoscale effect with no counterpart in continuum plate theory. Figure 4 compares the chirality-induced relative frequency deviation δ(L) for modes 1–4 as a function of sheet size. All four curves decrease monotonically with increasing sheet size, while the threshold sizes corresponding to the 1% criterion shift to larger values for higher-order modes.

**Table 2:** Mode 2 natural frequency and chirality deviation.

L (nm)	f_ZZ_ (GHz)	f_AC_ (GHz)	Δf (GHz)	δ (%)
2.0	1364.32	1229.23	135.09	10.99%
4.0	355.28	336.05	19.23	5.72%
8.0	88.82	86.4	2.42	2.80%
12.0	39.01	38.31	0.7	1.82%
16.0	22.21	21.93	0.28	1.25%
18.5	16.59	16.40	0.19	1.15%
24.5 (L*)	9.47	9.38	0.09	0.96%
30.0	6.32	6.29	0.03	0.47%

**Table 3:** Mode 3 natural frequency and chirality deviation.

L (nm)	f_ZZ_ (GHz)	f_AC_ (GHz)	Δf (GHz)	δ (%)
2.0	2273.81	1887.26	386.55	20.48%
6.0	252.65	231.42	21.23	9.17%
10.0	90.95	85.95	5	5.82%
12.0	63.16	60.38	2.78	4.60%
18.5	26.56	25.84	0.72	2.79%
24.0	15.79	15.54	0.25	1.61%
28.0 (L*)	11.6	11.49	0.11	0.96%
30.0	10.11	10.03	0.08	0.80%

**Table 4:** Mode 4 natural frequency and chirality deviation.

L (nm)	f_ZZ_ (GHz)	f_AC_ (GHz)	Δf (GHz)	δ (%)
2.0	2842.25	2444.34	397.91	16.28%
6.0	315.81	294.97	20.84	7.07%
10.0	113.69	108.8	4.89	4.49%
12.0	78.95	76.27	2.68	3.51%
18.5	33.21	32.55	0.66	2.03%
24.5	18.93	18.66	0.27	1.45%
30.0	12.63	12.51	0.12	1.08%
31.5 (L*)	11.46	11.35	0.11	0.97%

**Figure 5:**
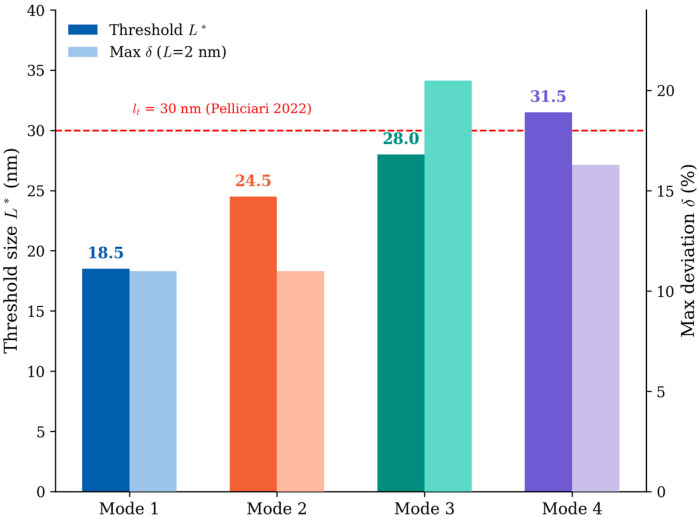
Threshold size L* as a function of vibration mode number (solid bars, left axis) and maximum chirality deviation at L = 2 nm (light-shaded bars, right axis). The red dashed horizontal line marks the in-plane threshold l_t_ = 30 nm reported by Pelliciari et al. [16] for reference.

The threshold size L* increases monotonically with mode number, as summarized in Table 5. Figure 5 provides a concise summary of the threshold size L* for each mode together with the maximum chirality deviation at L = 2 nm. It can be seen that the threshold size generally increases with mode number and becomes comparable to, or slightly higher than, the in-plane threshold l_t_ = 30 nm for the higher modes.

### 3.4. Mode Shape Analysis

**Figure 6:**
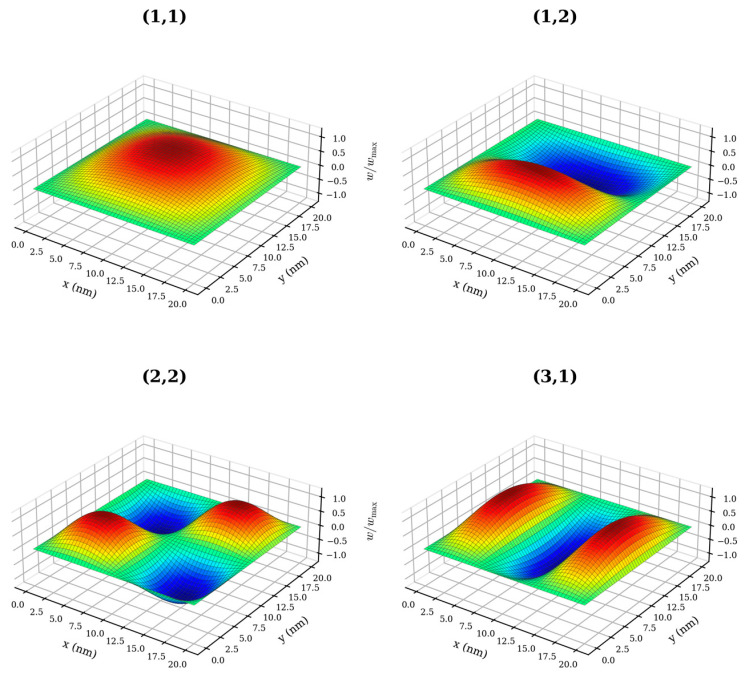
Normalized out-of-plane displacement mode shapes (w/w_max_) for the first four vibration modes of a simply supported square graphene sheet at L = 20 nm. Modes correspond to (m, n) = (1, 1), (1, 2), (2, 2), and (3, 1). Rainbow coloring from blue (w/w_max_ = −1) to red (w/w_max_ = +1).

Figure 6 presents the computed mode shapes for modes 1 through 4, displayed as normalized out-of-plane displacement plots with rainbow coloring from blue (w/w_max_ = −1) to red (w/w_max_ = +1). The mode shapes are consistent with the analytical solutions for a plate with SSSS boundary conditions.

Mode 1 (1, 1) exhibits a single half-wave in both the x- and y-directions, forming a smooth dome-like profile with maximum displacement at the sheet center. Mode 2 (1, 2) has one half-wave in x and two in y, producing one positive and one negative lobe separated by a nodal line at y = L/2. Mode 3 (2, 2) has two half-waves in both directions, resulting in a checkerboard pattern of four lobes with nodal lines at x = L/2 and y = L/2. Mode 4 (3, 1) has three half-waves in x and one in y, generating three alternating lobes along the x-direction with nodal lines at x = L/3 and x = 2L/3.

The spatial structure of each mode shape has a direct consequence for chirality sensitivity. Mode 1 distributes its bending strain energy broadly across the sheet interior, with only a small fraction residing near the edges, resulting in the weakest chirality sensitivity. The chirality deviation of mode 2 remains nearly identical to that of mode 1 across the entire size range (e.g., 10.99% vs. 10.98% at L = 2 nm); the underlying mechanism warrants further investigation. Mode 3, with half-wavelengths of L/2 in both directions simultaneously, concentrates bending curvature near all four edges, maximizing the relative contribution of chirality-dependent edge bonds to the total strain energy. This makes mode 3 the most chirality-sensitive among the first four modes at every sheet size investigated. Mode 4 has a shorter half-wavelength of L/3 in x but retains the full half-wavelength L in y; the enhanced edge sensitivity along x is partially offset by the weak edge contribution along y, giving δ_4_ < δ_3_ at all sizes. This interplay between two-dimensional mode shape geometry and discrete bond orientations at ZZ and AC edges is a purely nanoscale effect with no counterpart in continuum plate theory.

At small sheet sizes (L ≈ 2 nm), the mode shapes deviate from the smooth sinusoidal profiles of continuum plate theory. The discrete lattice imposes a minimum spatial resolution of approximately 2a ≈ 0.28 nm, so modes with half-wavelengths approaching this limit cannot be faithfully represented. For mode 4 at L = 2 nm, the shortest half-wavelength is L/3 ≈ 0.67 nm, corresponding to only about 4.7 bond lengths. In this regime, the continuum mode shape is a poor approximation of the actual discrete displacement pattern, and the distinction between ZZ and AC configurations arises as much from the lattice geometry as from macroscopic chirality.

### 3.5. Frequency Ratios and Comparison with Plate Theory

Table 6 presents the inter-mode frequency ratios f_n_/f_1_ for the AC configuration at selected sheet sizes, compared with the analytical Kirchhoff plate theory predictions for a square simply supported plate. For such a plate, the natural frequency of mode (m, n) scales as (m^2^ + n^2^), giving the theoretical ratios f_2_/f_1_ = 2.50 for mode (1, 2)/(2, 1), f_3_/f_1_ = 4.00 for mode (2, 2), and f_4_/f_1_ = 5.00 for mode (3, 1)/(1, 3).

At large sizes (L = 30 nm), the MM frequency ratios f_2_/f_1_ ≈ 2.50, f_3_/f_1_ ≈ 3.99, and f_4_/f_1_ ≈ 4.98 are in excellent agreement with the CT predictions, confirming that the MM model correctly recovers the continuum limit for large sheets. At small sizes (L = 2 nm), the ratios are all noticeably lower than the corresponding CT values. This indicates that higher-order modes are more strongly affected by chirality and lattice discreteness than mode 1, so their frequencies are reduced by a greater proportion relative to the continuum prediction. The deviation is most pronounced for mode 3 (2, 2), whose mode shape concentrates bending energy near all four edges simultaneously, amplifying the chirality-dependent edge-bond contribution as discussed in Section 3.3.

The deviation of frequency ratios from CT values at small sizes serves as a chirality-independent experimental criterion: if the measured ratios of the first four modes satisfy the (m^2^ + n^2^) scaling within a specified tolerance, the device can be considered to operate in the continuum regime; otherwise, discreteness corrections must be incorporated into the frequency calibration of multi-harmonic sensing protocols.

The convergence of inter-mode frequency ratios to their CT values as L increases provides an independent validation of the threshold size concept: it is only for L > L* that both the absolute frequencies and the relative frequency structure match the continuum plate theory predictions.

### 3.6. Design Guidelines for Graphene NEMS Resonators

The results of this study provide the following quantitative guidelines for the design of graphene NEMS resonators based on continuum plate theory:

(1) Single-mode devices (mode 1 only): a characteristic dimension L > 18.5 nm ensures that the CT prediction of the resonant frequency deviates from the MM result by less than 1% due to chirality effects. For L < 18.5 nm, the chirality of the graphene lattice must be accounted for, and MM or MD simulations are required for accurate frequency prediction.
(2) Dual-mode devices (modes 1–2): the minimum characteristic dimension is L > 24.5 nm. Multi-modal mass sensors that use two resonant frequencies to simultaneously determine the adsorbed mass and its position on the resonator must satisfy this criterion [26].
(3) Four-mode devices (modes 1–4): the minimum characteristic dimension is L > 31.5 nm. This criterion applies to advanced multi-modal sensing protocols that exploit higher harmonics.
(4) Frequency calibration: when using continuum theory to calibrate the frequencies of sub-threshold devices, the calibration error increases systematically with mode number. For a device at L = 18.5 nm, mode 1 is already calibrated to within 1% but mode 4 still carries a chirality-induced error of up to 2.0%. This systematic trend should be incorporated into the data processing pipeline of multi-modal sensing protocols.

## 4. Conclusions

The size-dependent out-of-plane vibrational behavior of graphene NEMS resonators has been systematically investigated using a molecular mechanics finite element approach. The C–C bonds are modeled as Euler–Bernoulli beam elements with bending stiffness derived from the bond-angle potential, and the natural frequencies of the first four modes are computed for square ZZ and AC graphene sheets under simply supported boundary conditions over the size range L = 2–31.5 nm. The main conclusions are as follows:

(1) Thenatural frequencies of both ZZ and AC configurations scale approximately as f ∝ 1/L^2^ in agreement with Kirchhoff plate theory, spanning two orders of magnitude across the investigated size range. The dimensionless frequency ratio Ω = f_MM_/f_CT_ is less than unity at small sizes due to edge-softening effects, and converges monotonically toward 1 with increasing sheet size, reaching 0.984 at L = 30 nm.
(2) Chirality-induced frequency deviations are significant at small sizes, reaching 10.98% for mode 1 and 20.48% for mode 3 at L = 2 nm. The deviation decreases monotonically with increasing L and vanishes asymptotically for large sheets.
(3) The threshold sizes determined by the 1% convergence criterion are L* = 18.5, 24.5, 28.0, and 31.5 nm for modes 1 through 4, respectively. Higher modes are more sensitive to chirality because their mode shapes concentrate energy near edges where the ZZ and AC bond environments differ, requiring larger sheet sizes for convergence.
(4) The mode 1 threshold L* = 18.5 nm is somewhat smaller than the in-plane threshold l_t_ = 30 nm reported by Pelliciari et al. [17], consistent with the fact that out-of-plane bending is governed exclusively by the bond-angle potential kθ, which is more spatially localized than the combined in-plane stiffness that also involves bond stretching.
(5) At large sizes, the inter-mode frequency ratios converge to the continuum plate theory values, providing independent validation that the MM model correctly recovers the continuum limit.
(6) Quantitative size criteria for graphene NEMS resonator design are established: L > 18.5 nm for reliable mode-1 predictions, L > 24.5 nm for modes 1–2, and L > 31.5 nm for all four modes. These thresholds directly inform the minimum device dimensions for which isotropic continuum models are applicable.

Future work may extend this study in the following directions: examination of the effect of lattice defects on threshold sizes; investigation of other boundary conditions such as clamped edges; extension to the large-amplitude nonlinear vibration regime to assess the chirality dependence of the Duffing nonlinearity coefficient; and application to graphene-based bio-NEMS where the frequency shifts of multiple modes are used for mass and position sensing; and experimental validation of the predicted size effects and chirality-dependent frequency deviations using advanced nanomechanical measurement techniques.

## Figures and Tables

**Table 1:** Mode 1 natural frequency and chirality deviation.

L (nm)	f_ZZ_ (GHz)	f_AC_ (GHz)	Δf (GHz)	δ (%)
2.0	568.45	512.18	56.27	10.98%
4.0	142.11	134.42	7.69	5.72%
6.0	63.16	60.85	2.31	3.80%
8.0	35.53	34.56	0.97	2.80%
10.0	22.74	22.21	0.53	2.38%
12.0	15.79	15.51	0.28	1.82%
14.0	11.6	11.43	0.17	1.51%
16.0	8.88	8.77	0.11	1.25%
18.5 (L*)	6.64	6.58	0.06	0.91%
21.0	5.15	5.11	0.04	0.81%
24.0	3.94	3.92	0.02	0.62%
27.0	3.11	3.1	0.01	0.49%
30.0	2.53	2.52	0.01	0.28%

**Table 5:** Summary of threshold sizes for modes 1–4 (δ = 1% criterion).

Moden	L* (nm)	Max δ (L = 2 nm)
1	18.5	10.98%
2	24.5	10.99%
3	28	20.48%
4	31.5	16.28%

**Table 6:** Inter-mode frequency ratios fn/f_1_ for AC configuration compared with continuum theory.

L (nm)	f_2_/f_1_	f_3_/f_1_	f_4_/f_1_	CT f_2_/f_1_	CT f_3_/f_1_	CT f_4_/f_1_
2.0	2.40	3.68	4.77	2.50	4.00	5.00
6.0	2.44	3.80	4.85	—	—	—
12.0	2.47	3.89	4.92	—	—	—
18.5	2.48	3.93	4.95	—	—	—
30.0	2.50	3.99	4.98	2.50	4.00	5.00

## Data Availability

The original contributions presented in this study are included in the article. Further inquiries can be directed to the corresponding author.
